# Correction: *Women's knowledge and attitudes surrounding abortion in Zambia: a crosssectional survey across three provinces*

**DOI:** 10.1136/bmjopen-2015-010076corr1

**Published:** 2016-10-06

Cresswell JA, Schroeder R, Dennis M, *et al*. Women's knowledge and attitudes surrounding abortion in Zambia: a crosssectional survey across three provinces. *BMJ Open* 2016;6:e010076. This paper has been resupplied with the CC BY license.

